# PhyloNet: a software package for analyzing and reconstructing reticulate evolutionary relationships

**DOI:** 10.1186/1471-2105-9-322

**Published:** 2008-07-28

**Authors:** Cuong Than, Derek Ruths, Luay Nakhleh

**Affiliations:** 1 Department of Computer Science, Rice University, 6100 Main Street, MS 132, Houston, TX, USA

## Abstract

**Background:**

Phylogenies, i.e., the evolutionary histories of groups of taxa, play a major role in representing the interrelationships among biological entities. Many software tools for reconstructing and evaluating such phylogenies have been proposed, almost all of which assume the underlying evolutionary history to be a tree. While trees give a satisfactory first-order approximation for many families of organisms, other families exhibit evolutionary mechanisms that cannot be represented by trees. Processes such as horizontal gene transfer (HGT), hybrid speciation, and interspecific recombination, collectively referred to as *reticulate evolutionary events*, result in *networks*, rather than trees, of relationships. Various software tools have been recently developed to analyze reticulate evolutionary relationships, which include SplitsTree4, LatTrans, EEEP, HorizStory, and T-REX.

**Results:**

In this paper, we report on the PhyloNet software package, which is a suite of tools for analyzing reticulate evolutionary relationships, or *evolutionary networks*, which are rooted, directed, acyclic graphs, leaf-labeled by a set of taxa. These tools can be classified into four categories: (1) evolutionary network representation: reading/writing evolutionary networks in a newly devised compact form; (2) evolutionary network characterization: analyzing evolutionary networks in terms of three basic building blocks – trees, clusters, and tripartitions; (3) evolutionary network comparison: comparing two evolutionary networks in terms of topological dissimilarities, as well as fitness to sequence evolution under a maximum parsimony criterion; and (4) evolutionary network reconstruction: reconstructing an evolutionary network from a species tree and a set of gene trees.

**Conclusion:**

The software package, PhyloNet, offers an array of utilities to allow for efficient and accurate analysis of evolutionary networks. The software package will help significantly in analyzing large data sets, as well as in studying the performance of evolutionary network reconstruction methods. Further, the software package supports the proposed eNewick format for compact representation of evolutionary networks, a feature that allows for efficient interoperability of evolutionary network software tools. Currently, all utilities in PhyloNet are invoked on the command line.

## Background

A phylogenetic tree models the evolutionary history of a set of taxa from their most recent common ancestor. The assumptions of strict divergence and vertical inheritance render trees appropriate for modeling the evolutionary histories of several groups of species or organisms. However, when *reticulate *evolutionary events such as horizontal gene transfer or interspecific recombination occur, the evolutionary history is more appropriately modeled by an evolutionary network.

Evidence of reticulate evolution has been shown in various domains in the Tree of Life. Bacteria obtain a large proportion of their genetic diversity through the acquisition of sequences from distantly related organisms, via horizontal gene transfer (HGT) [1]. Furthermore, more evidence of widespread HGT in plants is emerging recently [2-4]. Interspecific recombination is believed to be ubiquitous among viruses [5,6], and hybrid speciation is a major evolutionary mechanisms in plants, and groups of fish and frogs [7-10]. All of these processes result in networks, rather than trees, of evolutionary relationships, even though at the gene level evolutionary histories may be treelike, as we now describe.

**Figure 1:**
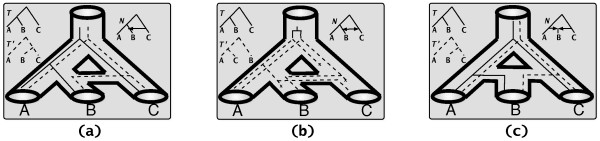
**Evolutionary networks and gene trees**. Gene trees *T *and *T*' within species networks *N*. (a) The gene whose tree is depicted with a dashed line is transferred from the genome of species *C *to that of species *B*. (b) Species *B *and *C *exchanged the two genes whose trees are *T *and *T*'. (c) Species *B *is a hybrid whose two parents are species *A *and *C*; each gene in the genome of species *B *has an evolutionary tree that is either *T *or *T*'.

Figure 1 illustrates the three major events that result in networks of evolutionary relationships among species, namely horizontal gene transfer, interspecific recombination, and hybrid speciation. The tubes depict the evolutionary network of the species, within which two gene trees are shown. In each box, the two possible gene trees *T *and *T*' are shown separately, as well as the network *N *at an abstract level. In an evolutionary scenario involving horizontal transfer, certain sites (specified by a specific substring within the DNA sequence of the species into which the horizontally transferred DNA was inserted) are inherited from another species (the tree *T*' in dashed lines in Figure 1(a)), while all others are inherited from the parent (the tree *T *in solid lines in Figure 1(a)). Thus, each site evolves down one of the trees contained inside the network.

In the case of interspecific recombination, as illustrated in Figure 1(b), some genetic material is exchanged between pairs of species; in this example, species *B *and *C *exchange genetic material. The genes involved in this exchange have an evolutionary history (gene tree *T*') that is different from that of the genes that are vertically transmitted (gene tree *T*).

In the case of hybrid speciation, as illustrated in Figure 1(c), the two parents contribute equally to the genetic material of the hybrid: in *diploid *hybridization, each parent contributes a single copy of each of its chromosomes, while in *polyploid *hybridization, each parent contributes all copies of its chromosomes. Thus, each set of "orthologous sites" from all taxa has an evolutionary history that is depicted by one of the trees inside the network.

A few software tools for analyzing reticulate evolutionary relationships have been developed recently. The SplitsTree4 tool, which incorporates several algorithms that have been developed by Daniel Huson and his co-workers, is a tool for reconstructing and visualizing splits networks [11]. The tool enables constructing networks from several types of data, including sequence data, distance matrices, and sets of trees. Two major differences exist between SplitsTree4 and PhyloNet. First, SplitsTree4 constructs and analyzes splits networks, which are graphical models of incompatibility in the data, whereas PhyloNet constructs and analyzes evolutionary networks, which are rooted, directed, acyclic graphs, that represent evolutionary relationships. Second, the two tools differ in the utilities they provide, and we view them as complementary. While SplitsTree4 is mainly aimed at reconstructing networks, PhyloNet has several utilities for evaluating networks.

Programs such as EEEP [12], HorizStory [13], LatTrans [14], and T-REX [15] are aimed at detecting horizontal gene transfer by reconciling a pair of species/gene trees. The PhyloNet software package that we developed contains an extended implementation of the RIATA-HGT algorithm [16] with several improved algorithmic techniques for computing multiple solutions and handling non-binary trees [17]. The new version of RIATA-HGT significantly outperforms, in terms of speed, EEEP, HorizStory and LatTrans, and performs at least as well in terms of accuracy [17,18]. We have recently added a new heuristic for inferring the support of HGT moves from bootstrap values of gene tree edges. Further, we have added the capability of visualizing the networks computed by RIATA-HGT. Besides RIATA-HGT, the PhyloNet software package implements methods for comparing and characterizing evolutionary networks, which include: (1) evolutionary network representation: reading/writing evolutionary networks in a newly devised compact form; (2) evolutionary network characterization: analyzing evolutionary networks in terms of three basic building blocks – trees, clusters, and tripartitions; (3) evolutionary network comparison: comparing two evolutionary networks in terms of topological dissimilarities, as well as fitness to sequence evolution under a maximum parsimony criterion; and (4) evolutionary network reconstruction: reconstructing an evolutionary network from a species tree and a set of gene trees. Furthermore, since various evolutionary network utilities use functionalities from the phylogenetic trees domain, PhyloNet provides a set of standalone phylogenetic tree analysis tools.

## Results and discussion

### The evolutionary network model

In this paper, we assume the "evolutionary network" model, which was formulated independently by Moret *et al*. [19] and Baroni *et al*. [20]. We now describe the model as well as some basic definitions and notations that we will use later.

Let *T *= (*V*, *E*) be a tree, where *V *and *E *are the *tree nodes *and *tree edges*, respectively, and let *L*(*T*) denote the tree's leaf set. Further, let *χ *be a set of taxa (organisms). Then, *T *is a *phylogenetic tree *over *χ *if there is a bijection between *χ *and *L*(*T*). Henceforth, we will identify the taxa set with the leaves they are mapped to, and let [*n*] = {1,..., *n*} denote the set of leaf-labels. A tree *T *is said to be *rooted *if the set of edges *E *is directed and there is a single node *r *∈ *V *with in-degree 0. Let *T *be a phylogenetic tree on set *χ *of taxa, and let *χ*' ⊆ *χ *be a subset of taxa; then, we denote by *T*|*χ*' the subtree with minimum number of nodes and edges that spans the leaves in *χ*' (in other words, *T*|*χ*' is the tree *T *restricted to subset *χ*' of its leaves).

**Figure 2:**
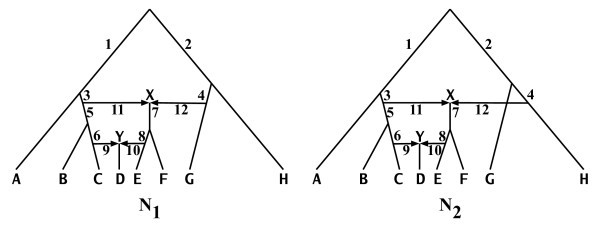
**Sample evolutionary networks**. Two evolutionary networks *N*_1 _and *N*_2_, each with eight leaves (labeled *A*,..., *H*) and two network nodes *X *and *Y*. Shown are the orientation of the network edges; all other edges are directed away from the root (toward the leaves) Notice that the difference between the two networks is that node *X *in *N*_1 _has lineage *G *as one of its parents, whereas node *X *in *N*_2 _has lineage *H *as one of its parents.

An evolutionary (phylogenetic) network *N *= (*V*, *E*) over the set *χ *of taxa is a rooted, directed, acyclic graph such that there is a bijection between *χ *and the set *L*(*N*) of the network's leaves (see Figure 2). The set *V *is partitioned into two sets: *V*_*T*_, the set of *tree nodes*, which are the nodes with in-degree smaller than two, and *V*_*N*_, the set of *network nodes*, which are the nodes with in-degree greater than or equal to two. Similarly, the set *E *is partitioned into two sets: *E*_*T*_, the set of *tree edges*, which are edges incident into tree nodes, and *E*_*N*_, the set of *network edges*, which are the edges incident into network nodes.

For two nodes *u *and *v *in directed graph *G*, we say that *v *is reachable from *u*, denoted by u⇝v if there exists a directed path from *u *to *v *in the tree *G*. For three nodes *u*, *v *and *x *in directed graph *G*, we write $u{⇝}^{\left[x\right]}v$ if all directed paths from *u *to *v *go through node *x*; $u/{⇝}^{\left[x\right]}v$ if no directed paths from *u *to *v *go through node *x*; and $u{⇝}^{\left[\hat{x}\right]}v$ if at least one directed path from *u *to *v *goes through node *x *and at least one directed path from *u *to *v *does not go through node *x*. For example, in network *N*_1 _in Figure 2, rooted at node r_1_, we have $r_{1}{⇝}^{\left[Y\right]}D$, $r_{1}/{⇝}^{\left[Y\right]}E$, and $r_{1}{⇝}^{\left[\hat{X}\right]}D$.

### Evolutionary network representation

**Figure 3:**
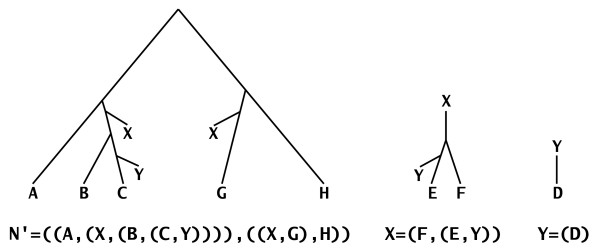
**A modified Newick format**. Three trees, *N*', *X*, and *Y*, along with their Newick representation. These three trees form the tree decomposition F of the evolutionary network *N*_1 _in Figure 2. The eNewick representation of *N *is the triplet ⟨*N*'; *X*; *Y*⟩.

The Newick format for representing and storing phylogenetic trees was adopted in 1986 [21], and it has been the standard for almost all phylogeny software packages ever since. This format captures an elegant correspondence between leaf-labeled trees and matched parentheses, where the leaves are represented by their names and the internal nodes by a matched pair of parentheses that contains a list of the Newick representation of all its children. Shown in Figure 3 are three trees along with their representations in the Newick format.

**Figure 4:**
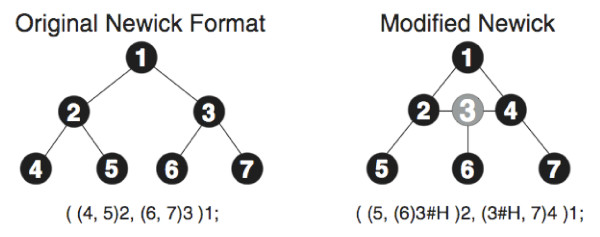
**The eNewick format**. A modified Newick format for representing evolutionary networks. The figure is taken from the paper by Morin *et. al*. [22].

Existing phylogenetic network software tools store these networks as adjacency lists of their underlying graphs, which are usually very large and necessitate translation of representations among the different tools. Morin and Moret [22] proposed a modified version of the Newick format for representing such networks. In their format, network nodes are represented by nodes labeled with #H, and those nodes are considered as two separate nodes in the normal Newick format for trees. See Figure 4 for an example. We have independently proposed a new method of *tree decomposition *of evolutionary networks, which provides the basis for a new format, *extended Newick *(or eNewick for short), and used it as a compact representation of evolutionary networks. The idea in our method is to break the network into a set of trees, and then represent the network as a collection of Newick representations of those trees. Since the eNewick format is nothing but a collection of trees in the Newick format, it follows that eNewick can represent unrooted networks. However, both in this paper as well as in the PhyloNet utilities, rooting is assumed, since different ways of rooting the same evolutionary networks may imply different evolutionary relationships.

Let *N *= (*V *= (*V*_*N *_∪ *V*_*T*_); *E*) be an evolutionary network, with |*V*_*N*_| = ℓ. We create a forest of ℓ trees as follows.

• For every *u*_*i *_∈ *V*_*N*_

- Compute the set *V*_*i *_= {*v *∈ *V *: (*v*, *u*_*i*_) ∈ *E*} of *u*_*i*_'s parents;

- Create *k *new leaves, all labeled with *x*_*i *_({*x*_*i*_} ∩ *L*(*N*) = ∅);

- Delete from *V *the set of all edges in *V*_*i *_× {*u*_*i*_};

- Add to *V *the set of edges *V*_*i *_× {*x*_*i*_};

- Assign *x*_*i *_as the name of the tree rooted at node *u*_*i*_;

The result is a forest of trees F = {*t*_1_,..., *t*_ℓ_} such that (1) |*L*(*t*_*i*_)| ≥ 1 for every 1 ≤ *i *≤ ℓ, (2) $\cup_{i=1}^{\ell }L(t_{i})=L(N)$ and (3) *L*(*t*_*i*_) ∩ *L*(*t*_*j*_) = ∅ for every 1 ≤ *i*, *j *≤ ℓ and *i *≠ *j*. We call F the *tree decomposition *of *N*. Then, the eNewick representation of *N *is the ℓ-tuple ⟨*n*(*t*_1_);...; *n*(*t*_ℓ_)⟩, where *n*(*t*_*i*_) is the Newick representation of tree *t*_*i*_. Figure 3 shows the tree decomposition and eNewick representation of the network *N*_1 _in Figure 2.

In the case of modeling networks with horizontal gene transfer events, it is often very helpful to the biologist to know what the species tree is and what the additional set of HGT events are. Such information is "lost" in an eNewick representation, unless the representation is extended further to keep a record of the "species tree parent" of each network node. Therefore, in this case (which is the output of RIATA-HGT) we opt for the format of a species tree *T*, in Newick format, followed by a list of the HGT edges, each written as *X *→ *Y*, where *X *and *Y *are two nodes in *T*.

### Evolutionary network characterization

**Figure 5:**
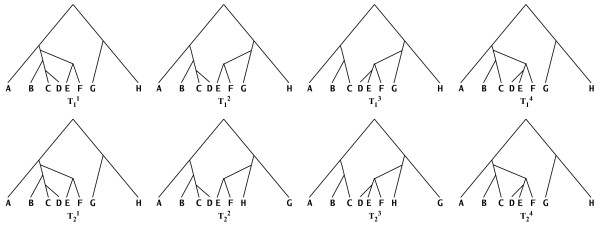
**Trees within networks**. The sets $T(N_{1})=\{T_{1}^{1},T_{1}^{2},T_{1}^{3},T_{1}^{4}\}$ and $T(N_{2})=\{T_{2}^{1},T_{2}^{2},T_{2}^{3},T_{2}^{4}\}$ of all eight trees induced by the two networks *N*_1 _and *N*_2_, respectively, in Figure 2.

As we described in the background section, an evolutionary network induces, or contains, a set of trees. We now formalize this concept and characterize networks in terms of the trees they induce. A tree *T *is induced by a network *N *if *T *is obtained from *N *as follows: (1) for each node of in-degree larger than one, remove all but one of the network edges incident into it, and (2) for every node of in-degree and out-degree 1, and whose parent is *u *and child is *v*, remove the two edges incident with it, and add an edge from *u *to *v*. We denote by T(*N*) the set of all trees induced by *N*. Figure 5 shows the sets T(*N*_1_) and T(*N*_2_) for the two networks *N*_1 _and *N*_2 _in Figure 2. It is important to note that this set of trees is completely different from the set of trees obtained by the tree decomposition we introduced to facilitate the eNewick format. An evolutionary network *N *with *V*_*N *_= {*v*_1_,..., *v*_ℓ_}, such that *indegree*(*v*_*i*_) = *ρ*_*i*_, induces *m *trees, where $m\leq \prod_{i=1}^{\ell }\rho_{i}$

Given an evolutionary network *N*, the set T(*N*) is unique. Further, this set informs about the possible gene histories that the network reconciles.

In addition to characterizing evolutionary networks by the set of trees they induce, we consider a *cluster*-based characterization. This view of evolutionary networks is very important for understanding the relationships among the "evolutionary perspective" of evolutionary networks and the "cluster, or splits, perspective", which is adopted in various methods [23,24]. Let *T *= (*V*, *E*) be a phylogenetic tree on set *χ *of taxa and rooted at node *r*. Each edge *e *= (*u*, *v*) ∈ *E *induces a *cluster *of taxa, denoted *c*_*e*_, which is the set $\left\{x\in \chi :r{⇝}^{\left[v\right]}x\right\}$. The (nontrivial) clusters of tree *T *is the set C(*T*) = {*c*_*e *_: *e *is an internal edge in *E*}. The topology of a tree is a compact graphical representation of its clusters, where the root of the clade that corresponds to cluster *c*_*e*' _lies on the path from the root of the tree to the root of the clade that corresponds to cluster *c*_*e *_if and only if *c*_*e *_⊆ *c*_*e*'_. Hence, clusters play an important role in phylogenetic tree characterization and reconstruction. A straightforward way to extend this concept to evolutionary networks is to define the set of clusters of evolutionary network *N *as $C(N)=\cup_{T\in T(N)}C(T)$. The clusters of the two networks *N*_1 _and *N*_2 _in Figure 2 are listed in Table 1.

**Table 1:** The clusters of the two networks in Figure 2.

Network *N*_1_	Network *N*_2_
{*B*, *C*}	{*B*, *C*}
{*C*, *D*}	{*C*, *D*}
{*B*, *C*, *D*}	{*B*, *C*, *D*}
{*D*, *E*}	{*D*, *E*}
{*E*, *F*}	{*E*, *F*}
{*D*, *E*, *F*}	{*D*, *E*, *F*}
{*B*, *C*, *D*, *E*, *F*}	{*B*, *C*, *D*, *E*, *F*}
{*A*, *B*, *C*}	{*A*, *B*, *C*}
{*A*, *B*, *C*, *D*}	{*A*, *B*, *C*, *D*}
{*A*, *B*, *C*, *D*, *E*, *F*}	{*A*, *B*, *C*, *D*, *E*, *F*}
{**E**, **F**, **G**}	{**E**, **F**, **H**}
{**D**, **E**, **F**, **G**}	{**D**, **E**, **F**, **H**}
{*G*, *H*}	{*G*, *H*}
{*D*, *E*, *F*, *G*, *H*}	{*D*, *E*, *F*, *G*, *H*}

A table of the (nontrivial) clusters of the two networks *N*_1 _and *N*_2 _in Figure 2, denoted by C(*N*_1_) and C(*N*_2_), respectively, in the text. Highlighted are rows corresponding to clusters that differ between the two networks.

In this form of cluster-based characterization, clusters are unweighted; equivalently, all clusters are weighted equally. One option of weighting the clusters is by considering the fraction of trees in which it appears. In other words, the weight of a cluster *c*_*e *_can be computed as

$$w(c_{e})=\frac{\left|\{T\in T(N):c_{e}\in C(T)\}\right|}{\left|T(N)\right|}.$$

This weighting scheme informs not only about the clusters of taxa that the network represents, but also how many gene trees in the input share each cluster. It is important to note here that this weighting of a cluster should not be confused with, or used in lieu of, support values of clusters, since a cluster may appear in only one gene tree and have a high support (e.g., by having a high bootstrap value on the edge that defines it) whereas a poorly supported cluster may appear in several trees.

Nakhleh and colleagues have recently introduced a new characterization of evolutionary networks based on the *tripartitions *of their edges [19]. Let *e *= (*u*, *v*) be an edge in an evolutionary network on set *χ *of taxa and rooted at node *r*. We define three disjoint sets *A*_*e *_= $\left\{x\in \chi :r{⇝}^{\left[v\right]}x\right\}$, *B*_*e *_= $\left\{x\in \chi :r{⇝}^{\left[\hat{v}\right]}x\right\}$, and *C*_*e *_= $\left\{x\in \chi :r/{⇝}^{\left[v\right]}x\right\}$. Then, the tripartition induced by edge *e*, denoted *θ*_*e*_, is the triplet ⟨*A*_*e*_; *B*_*e*_; *C*_*e*_⟩. Roughly speaking, the tripartition induced by an edge is the three sets of taxa reachable from the root only through that edge (*A*_*e*_), reachable through that edge but not exclusively (*B*_*e*_), and not reachable through that edge (*C*_*e*_). The set of (nontrivial) tripartitions induced by a evolutionary network *N*, denoted by *θ*(*N*), is {*θ*_*e *_: *e *is an internal edge in *E*}. The tripartitions of the two networks *N*_1 _and *N*_2 _in Figure 2 are listed in Table 2.

**Table 2:** The tripartitions of the two networks in Figure 2.

Edge Label	Network *N*_1_	Network *N*_2_
1	⟨{*A*, *B*, *C*}, {*D*, *E*, *F*}, {*G*, *H*}⟩	⟨{*A*, *B*, *C*}, {*D*, *E*, *F*}, {*G*, *H*}⟩
2	⟨{*G*, *H*}, {*D*, *E*, *F*}, {*A*, *B*, *C*}⟩	⟨{*G*, *H*}, {*D*, *E*, *F*}, {*A*, *B*, *C*}⟩
3	⟨{*B*, *C*}, {*D*, *E*, *F*}, {*A*, *G*, *H*}⟩	⟨{*B*, *C*}, {*D*, *E*, *F*}, {*A*, *G*, *H*}⟩
**4**	⟨{**G**}, {**D**, **E**, **F**}, {**A**, **B**, **C**, **H**}⟩	⟨{**H**}, {**D**, **E**, **F**}, {**A**, **B**, **C**, **G**}⟩
5	⟨{*B*, *C*}, {*D*}, {*A*, *E*, *F*, *G*, *H*}⟩	⟨{*B*, *C*}, {*D*}, {*A*, *E*, *F*, *G*, *H*}⟩
6	⟨{*C*}, {*D*}, {*A*, *B*, *E*, *F*, *G*, *H*}⟩	⟨{*C*}, {*D*}, {*A*, *B*, *E*, *F*, *G*, *H*}⟩
7	⟨{*E*, *F*}, {*D*}, {*A*, *B*, *C*, *G*, *H*}⟩	⟨{*E*, *F*}, {*D*}, {*A*, *B*, *C*, *G*, *H*}⟩
8	⟨{*E*}, {*D*}, {*A*, *B*, *C*, *F*, *G*, *H*}⟩	⟨{*E*}, {*D*}, {*A*, *B*, *C*, *F*, *G*, *H*}⟩
9	⟨{*D*}, {}, {*A*, *B*, *C*, *E*, *F*, *G*, *H*}⟩	⟨{*D*}, {}, {*A*, *B*, *C*, *E*, *F*, *G*, *H*}⟩
10	⟨{*D*}, {}, {*A*, *B*, *C*, *E*, *F*, *G*, *H*}⟩	⟨{*D*}, {}, {*A*, *B*, *C*, *E*, *F*, *G*, *H*}⟩
11	⟨{*E*, *F*}, {*D*}, {*A*, *B*, *C*, *G*, *H*}⟩	⟨{*E*, *F*}, {*D*}, {*A*, *B*, *C*, *G*, *H*}⟩
12	⟨{*E*, *F*}, {*D*}, {*A*, *B*, *C*, *G*, *H*}⟩	⟨{*E*, *F*}, {*D*}, {*A*, *B*, *C*, *G*, *H*}⟩

A table of the (nontrivial) tripartitions of the two networks *N*_1 _and *N*_2 _in Figure 2, denoted by *θ*(*N*_1_) and *θ*(*N*_2_), respectively, in the text. Highlighted are rows corresponding to tripartitions that differ between the two networks.

Tripartition-based characterization of an evolutionary network helps to identify clades across which no genetic transfer occurred. If *A*_*e *_= *X *and *B*_*e *_= ∅ for an edge *e *= (*u*, *v*), this implies that the clade rooted at node *v *has set *X *of leaves, and there does not exist any exchange or transfer of genetic material between any organism in *X *and another organism that is not in *X*. Equivalently, an evolutionary network can be partitioned into a collection {*N*_1_, *N*_2_,..., *N*_*k*_} of evolutionary networks that result from *N *by deleting every edge *e *for which *B*_*e *_= ∅. Such a partition informs about the "locality" of reticulation events: each event in *N *is local to one of the *k *components in {*N*_1_, *N*_2_,..., *N*_*k*_}. Further, this partition implies that each of the trees in T(*N*) has *k *clades that have the sets {*L*(*N*_1_), *L*(*N*_2_),..., *L*(*N*_*k*_)} of leaves.

### Evolutionary network comparison

Researchers are often interested in quantifying the similarities and differences between two phylogenies reconstructed either from two different sources of data or from two different reconstruction methods. Such a quantification provides insights into agreements and disagreements among analyses, confidence values for different parts of the phylogenies, and metrics for comparing the performance of phylogenetic reconstruction methods. In the context of phylogenetic trees, this quantification is most commonly done based on one of two criteria:

• *Topological differences*. The topologies, or shapes, of two phylogenetic trees are compared, and their differences are quantified. Several measures have been introduced to quantify topological differences and similarities between a pair of trees, such as the Robinson-Foulds measure and the SPR distance; see [25,26] for a description of several such measures.

• *Fitness to sequence evolution*. When two phylogenies are reconstructed from the same sequence data set, it is common to compare them in terms of how well they model the evolution of the sequences. The most commonly used criteria for measuring such fitness are maximum parsimony, maximum likelihood, and the Bayesian posterior probability; see [25] for a detailed discussion of all three criteria.

In this section, we report on the capabilities in PhyloNet for comparing two evolutionary networks in terms of their topological differences and similarities, as well as in terms of their fitness to sequence evolution based on the maximum parsimony criterion.

For quantifying the dissimilarity between two evolutionary network topologies *N*_1 _and *N*_2_, we want a measure *m*(·,·) that satisfies three conditions:

*Identity*: *m*(*N*_1_, *N*_2_) = 0 if and only if *N*_1 _and *N*_2 _are *equivalent*;

*Symmetry*: *m*(*N*_1_, *N*_2_) = *m*(*N*_2_, *N*_1_); and

*Triangle inequality*: *m*(*N*_1_, *N*_3_) + *m*(*N*_3_, *N*_2_) ≥ *m*(*N*_1_, *N*_2_) for any evolutionary network *N*_3_.

This issue of evolutionary network equivalence was discussed in [19]. The three characterizations of evolutionary networks that we described above induce three measures which we now define. Let *N*_1 _and *N*_2 _be two evolutionary networks on the same set *X *of leaves; we define the three measures as follows.

#### Tree-based comparison

**Figure 6:**
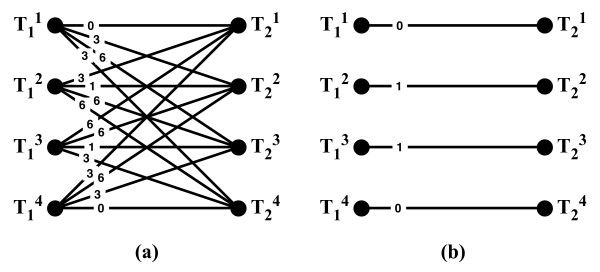
**Tree-based comparison of networks**. Illustration of the tree-based network comparison measure. (a) The weighted bipartite graph *G *that is constructed from the two networks *N*_1 _and *N*_2 _in Figure 2. On the left are four nodes that correspond to the four trees in T(*N*_1_) and on the right are four nodes that correspond to the four trees in T(*N*_2_). The weight of an edge between $T_{1}^{i}$ and $T_{2}^{j}$ is the values of the Robinson-Foulds (RF) distance between the two trees, which is computed as the number of clusters present in one but not both of the trees, divided by 2. (b) The edges that comprise the minimum-weight edge cover of the bipartite graph *G*. The weight of this cover is 2, which is the sum of the weights of the edges in the cover; therefore, *m*^*tree*^(*N*_1_, *N*_2_) = 2.

Let T(*N*_1_) and T(*N*_2_) be the two sets of all trees induced by the two networks, and let *d*(·,·) be a distance metric on trees (see [26] for examples of such metrics). The idea is to compare the two networks based on how similar their corresponding sets of trees are. We formalize this as follows. Construct a weighted complete bipartite graph *G*(*U*_1_, *U*_2_, *E*), where |*U*_*i*_| = |T(*N*_*i*_)|, and there are two bijections *f*_*i *_: *U*_*i *_→ T(*N*_*i*_) for *i *= 1, 2. The weight of an edge *e *= (*u*, *v*) ∈ *E *for *u *∈ *U*_1 _and *v *∈ *U*_2_, *w*(*e*) = *d*(*f*_1_(*u*), *f*_2_(*v*)). Then, the tree-based measure *m*^*tree*^(*N*_1_, *N*_2_) is defined as the weight of a minimum-weight edge cover of *G *divided by, the number of the edges in the cover. In its current implementation, PhyloNet uses the Robinson-Foulds distance measure [27] for *d*. The tree-based measure was first introduced by Nakhleh *et al*. [28]. An illustration of tree-based comparison of the two networks *N*_1 _and *N*_2 _in Figure 2 is given in Figure 6. Shown on the left of Figure 6 is the bipartite graph *G *built from the sets T(*N*_1_) and T(*N*_2_) of trees induced by the two networks; these two sets are shown in Figure 5. The weight of each edge connecting two nodes in *G *is the RF distance between the two trees corresponding to these two nodes. These weights can be normalized by the number of internal edges in the trees. Since each of the eight trees has six internal edges, the weight of each edge in *G *can be divided by six to normalize it.

Shown on the right of Figure 6 is the minimum-weight edge cover of *G*, which is the set of edges that satisfies two conditions: (1) each node in *G *must be the endpoint of at least one edge in the set, and (2) the sum of the weights of the edges in the set is minimum among all sets of edges satisfying condition 1. In this case, the four edges shown are a cover, since each node in *G *is "covered" by at least one edge (here, each node is covered by exactly one edge). Further, it is of minimum weight, which equals 2, since a simple inspection yields that every other cover has a weight larger than 2. Since the cover has four edges in it, we have *m*^*tree*^(*N*_1_, *N*_2_) = (0 + 0 + 1/6 + 1/6)/4 = 1/12. If we use the raw RF values, then *m*^*tree*^(*N*_1_, *N*_2_) = (0 + 0 + 1 + 1)/4 = 1/2.

#### Cluster-based comparison

Let *C*_1 _= C(*N*_1_) and *C*_2 _= C(*N*_2_) be the two sets of all clusters induced by the two networks. We define the measure based on these two sets to be

$$m^{cluster}(N_{1},N_{2})=\left(\frac{\left|C_{1}-C_{2}\right|}{\left|C_{1}\right|}+\frac{\left|C_{2}-C_{1}\right|}{\left|C_{2}\right|}\right)/2.$$

The rationale behind this measure is that it is the sum of the ratios of clusters present in one but not both networks. The cluster-based measure was first introduced by Nakhleh *et al*. [29]. The sets *C*_1 _= C(*N*_1_) and *C*_2 _= C(*N*_2_) of the two networks *N*_1 _and *N*_2 _in Figure 2 are listed in Table 1, with |*C*_1_| = |*C*_2_| = 14. Since |*C*_1 _- *C*_2_| = |*C*_2 _- *C*_1_| = 2 (the two highlighted clusters in Table 1), we have *m*^*cluster*^(*N*_1_, *N*_2_) = 1/7. A similar weighting scheme to that described in the previous section can be used to incorporate the fraction of trees in which a cluster appears into the measure calculation.

#### Tripartition-based comparison

Let *θ*_1 _= *θ*(*N*_1_) and *θ*_2 _= *θ*(*N*_2_) be the two sets of all tripartitions induced by the two networks. We define the measure based on these two sets to be

$$m^{tripartition}(N_{1},N_{2})=\left(\frac{\left|\theta_{1}-\theta_{2}\right|}{\left|\theta_{1}\right|}+\frac{\left|\theta_{2}-\theta_{1}\right|}{\left|\theta_{2}\right|}\right)/2.$$

This measure views the two networks in terms of the sets of edges they define (where an edge is in a 1-1 correspondence with a tripartition) and computes the sum of the ratios of edges present in one but not both networks. The tripartition-based measure was devised by Moret *et al*. [19]. The sets *θ*_1 _= *θ*(*N*_1_) and *θ*_2 _= *θ*(*N*_2_) of the two networks *N*_1 _and *N*_2 _in Figure 2 are listed in Table 2, with |*θ*_1_| = |*θ*_2_| = 12. Since |*θ*_1 _- *θ*_2_| = |*θ*_2 _- *θ*_1_| = 1 (the highlighted tripartition in Table 2), we have *m*^*tripartition*^(*N*_1_, *N*_2_) = 1/12.

#### Which measure to use?

Several distance measures, such as the Robinson-Fould measure and the Subtree Prune and Regraft (SPR) distance, have been introduced over the years to quantify the difference between the topologies of a pair of phylogenetic trees; e.g., see [25,26] for description of many of these measures. Even though these measures may compute different distance values on the same pair of trees, there has been no consensus as to which measure should be used in general [30]. It may be the case that the Robinson-Foulds measure is more commonly used than the others, but this may be a mere reflection of its very low time requirements as compared to the other, more compute-intensive, measures.

Regarding the three measures for comparing networks, a scenario analogous to that in phylogenetic trees arises here: each measure gives a different quantification of the dissimilarity between two networks based on one of the three ways to characterize a given network. As shown in the examples above, some or all of these measures may compute the same value for a given pair of networks, but that may not always be the case. Tree-based comparison of networks can be viewed as a method to quantify how similar, or dissimilar, two networks are in terms of their quality as a summary of a collection of trees. In some cases, even though two networks "look different," they may be identical in terms of the trees they induce – this is the issue of indistinguishability of networks from a collection of trees that Nakhleh and colleagues discussed in [19]. In such a case, using the tree-based comparison, or equivalently the cluster-based comparison, is most appropriate. However, if the similarity/dissimilarity of two networks means something close to an *isomorphism*, then the tripartition-based measure is more appropriate. However, it is important to note that none of the three measures described here is a metric on the general space of all evolutionary networks labeled by a given set of taxa.

A practical distinction among the three measures can be derived based on the methods used to infer the evolutionary history of the set of species under study. Methods such as SplitsTree [23] and NeighborNet [24] represent the evolutionary history as a set of splits, or clusters, hence making it more natural to use cluster-based comparison to study their performance. Methods such as RIATA-HGT [16] and LatTrans [14] compute evolutionary networks that are rooted, directed, acylic graphs, where internal nodes have an evolutionary implication in terms of ancestry. For these two methods, all three measures are appropriate. When the evolutionary history of a set of species is represented as a collection of its constituent gene trees, the tree-based measure is most appropriate.

Finally, a clear distinction can be made among the methods in terms of computational requirements. In their current implementations, the tripartition-based measure is very fast in practice, taking time that is polynomial in the size of the two networks. On the other hand, the tree- and cluster-based measures are much slower, taking time that is exponential in the number of network nodes in the two networks (since these measures compute explicitly all trees inside each of the two networks). In light of recent complexity results that we obtained [31], it is very likely that no polynomial-time algorithms exist for computing the tree- and cluster-based measures in general.

#### Parsimony of evolutionary networks

Nakhleh and colleagues have recently formalized a maximum parsimony (MP) criterion for evolutionary networks [32] and demonstrated its utility in reconstructing evolutionary networks on both biological and synthetic data sets [33]. In this section, we describe a PhyloNet utility that allows for comparing two evolutionary networks in terms of their fitness to the evolution of a sequence data set, based on the MP criterion. We first begin with a brief review of the MP criterion, based on the exposition in [32].

The relationship between an evolutionary network and its constituent trees, as described in the background section, is the basis for the MP extension to evolutionary networks.

**Definition 1 ***The Hamming distance between two equal-length sequences x and y, denoted by H*(*x*, *y*)*, is the number of positions j such that x*_*j *_≠ *y*_*j*_.

Given a fully-labeled tree *T*, i.e., a tree in which each node *v *is labeled by a sequence *s*_*v *_over some alphabet Σ, we define the Hamming distance of an edge *e *∈ *E*(*T*), denoted by *H*(*e*), to be *H*(*s*_*u*_, *s*_*v*_), where *u *and *v *are the two endpoints of *e*. We now define the parsimony score of a tree *T*.

**Definition 2 ***The parsimony score of a fully-labeled tree T, is Σ*_*e *∈ *E*(*T*) _*H*(*e*)*. Given a set S of sequences, a maximum parsimony tree for S is a tree leaf-labeled by S and assigned labels for the internal nodes, of minimum parsimony score*.

The parsimony definitions can be extended in a straightforward manner to incorporate different site substitution matrices, where different substitutions do not necessarily contribute equally to the parsimony score, by simply modifying the formula *H*(*x*, *y*) to reflect the weights. Let Σ be the set of states that the two sequences *x *and *y *can take (e.g., Σ = {*A*, *C*, *T*, *G*} for DNA sequences), and *W *the site substitution matrix such that *W*[*σ*_1_, *σ*_2_] is the weight of replacing *σ*_1 _by *σ*_2_, for every *σ*_1_, *σ*_2 _∈ Σ. In particular, the *identity *site substitution matrix satisfies *W*[*σ*_1_, *σ*_2_] = 0 when *σ*_1 _= *σ*_2_, and *W*[*σ*_1_, *σ*_2_] = 1 otherwise. The weighted Hamming distance between two sequence is *H*(*x*, *y*) = Σ_1 ≤ *i *≤ *k *_*W*(*x*_*i*_, *y*_*i*_), where *k *is the length of the sequences *x *and *y*. The rest of the definitions are identical to the simple Hamming distance case. As described above, the evolutionary history of a single (non-recombining) gene is modeled by one of the trees contained inside the evolutionary network of the species containing that gene. Therefore the evolutionary history of a site *s *is also modeled by a tree contained inside the evolutionary network. A natural way to extend the tree-based parsimony score to fit a dataset that evolved on a network is to define the parsimony score for each site as the minimum parsimony score of that site over all trees contained inside the network.

**Definition 3 ***(*[32]*) The parsimony score of a network N leaf-labeled by a set S of taxa, is*

$$NCost(N,S):=\sum_{s_{i}\in S}\left(\min_{T\in T(N)}TCost(T,s_{i})\right)$$

*where TCost*(*T*, *s*_*i*_) *is the parsimony score of site s*_*i *_*on tree T*.

Notice that as usually large segments of DNA, rather than single sites, evolve together, Definition 3 can be extended easily to reflect this fact, by partitioning the sequences *S *into non-overlapping blocks *b*_*i *_of sites, rather than sites *s*_*i*_, and replacing *s*_*i *_by *b*_*i *_in Definition 3. This extension may be very significant if, for example, the evolutionary history of a gene includes some recombination events, and hence that evolutionary history is not a single tree. In this case, the recombination breakpoint can be detected by experimenting with different block sizes.

The MP utility in PhyloNet allows the user to specify two evolutionary networks (either or both of which can be a tree) *N*_1 _and *N*_2 _and a sequence data set *S*, and computes the parsimony scores *NCost*(*N*_1_, *S*) and *NCost*(*N*_2_, *S*). The user can then compare the two scores and evaluate the fitness of the networks to the data set *S *based on the difference in the scores. Further, the utility allows the user, for example, to evaluate the significance of each network edge in a network *N *by comparing the parsimony scores of two different versions of *N *that contain different subsets of the network edges in *N*.

### Reconstructing evolutionary networks from species/gene trees

Assuming incongruence among gene and species trees is the result of HGT events only, the *Phylogeny-based HGT Reconstruction Problem*, or HGT Reconstruction Problem for short, is defined as follows:

**Problem 1 ***(HGT Reconstruction Problem)*

**Input: ***A species tree ST and a set *T = {*T*_1_,..., *T*_*p*_}*of gene trees*.

**Output: ***An evolutionary network N, obtained by adding a minimal set of edges Ξ to T, such that N contains every tree T*_*i *_∈ T

The minimization criterion is a reflection of Occam's razor: in the absence of any additional biological knowledge, HGT events should be used sparingly to explain data features otherwise explainable under a tree model. The problem of finding a minimum-cardinality set of HGT events whose occurrence on species tree *ST *would give rise to the gene trees in set T is computationally hard [34]. In [16], Nakhleh *et al*. introduced an accurate, polynomial-time heuristic, RIATA-HGT, for solving the HGT Reconstruction Problem for a pair of species and gene trees (in other words, RIATA-HGT currently handles the case where |T| = 1). In a nutshell, the method computes the maximum agreement subtree [35] of the species tree and each of the gene trees, and adds HGT edges to connect all subtrees that do not appear in the maximum agreement subtree. Theoretically, RIATA-HGT may not compute the minimum-cardinality set of HGT events; nonetheless, experimental results show very good empirical performance on synthetic as well as biological data [16].

#### Computing multiple solutions and the graphical output

RIATA-HGT was designed originally to compute a single solution to the problem, and was mainly aimed at binary trees. Later, Than and Nakhleh [17] extended the method to compute multiple solutions and to handle non-binary trees. These two features are very significant: the former allows biologists to explore multiple potential HGT scenarios, whereas the latter allows for analyzing trees in which some edges were contracted due to inaccuracies (see [36] for example). We have conducted an experimental study to compare the performance of RIATA-HGT with LatTrans [18]. Although RIATA-HGT and LatTrans [14] have almost the same performance in terms of the number of HGT solutions and the solution size, the former runs much faster than the latter.

For a compact representation of multiple solutions, we introduce four terms:

• An *event*: this is a single HGT edge, written in the form of *X *→ *Y*, where *X *and *Y *are two nodes in the species tree.

• A *subsolution*: this is an *atomic *set of events, which forms a part of an overall solution. In other words, either all or none of the events of a subsolution are taken in a solution.

• A *component*: a set of components and/or subsolutions. Two components at the same level of decomposition are independent, in that an element of each component is needed to form a solution.

• A *solution*: the union of a single element from each component at the highest level.

**Figure 7:**
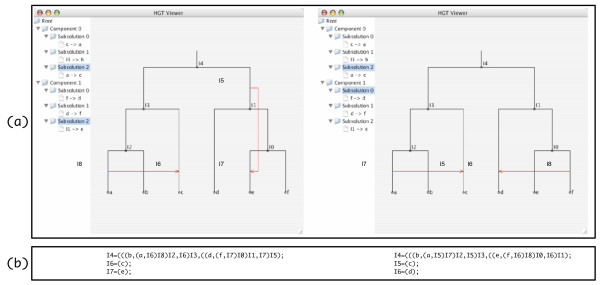
**Screenshot of the graphical output of RIATA-HGT**. (a) Screen captures of the graphical output of RIATA-HGT on the pair of trees (((*a*, *b*), *c*), (*d*, (*e*, *f*))) and (((*a*, *c*), *b*), ((*d*, *f*), *e*)). (b) The eNewick representations of the two selected networks.

To illustrate these concepts, consider species tree (((*a*, *b*), *c*), (*d*, (*e*, *f*))) and the gene tree (((*a*, *c*), *b*), ((*d*, *f*), *e*)). Observe, that each HGT event required to reconcile the two trees has both endpoints in the subtree ((*a*, *b*), *c*) or both endpoints in the subtree (*d*, (*e*, *f*)), and no HGT event has endpoints in both subtrees. In this case, RIATA-HGT divides the pair of trees into two pairs:

• Pair 1: ((*a*, *b*), *c*) and ((*a*, *c*), *b*)

• Pair 2: (*d*, (*e*, *f*)) and ((*d*, *f*), *e*),

and solves the HGT Reconstruction Problem on each of the two pairs *independently*. The set of solutions of each pair is a component. Notice that for each pair there are three possible ways to reconcile them; each such way is a called a subsolution. Each subsolution is a set of events, which in this case is only one event. Figure 7(a) shows the screen captures of two graphical outputs that correspond to two solutions on this pair of trees. Notice that if a component can be further divided into independent components, RIATA-HGT would do so, which will result in components at different levels, with the largest components being at the highest level.

The compact representation of RIATA-HGT's output in terms of subsolutions and components is especially helpful when the number of solutions is large. RIATA-HGT also has an option to display all complete solutions. RIATA-HGT enumerates all complete solutions that are compactly represented as described in the preceding paragraphs. Each solution, which is a set of HGT events, along with the species tree defines an evolutionary network, which RIATA-HGT displays in the eNewick format. For example, for the trees (((*a*, *b*), *c*), (*d*, (*e*, *f*))) and (((*a*, *c*), *b*), ((*d*, *f*), *e*)), RIATA-HGT outputs 9 different networks in the eNewick format, if RIATA-HGT's option for displaying complete solutions is on. Figure 7(b) shows the corresponding eNewick representations.

From the multiple comparisons between a species and a set of trees, RIATA-HGT offers a (strict) consensus network. For each pair of species tree and gene tree, RIATA-HGT computes a set of HGT events for reconciling them. To obtain the consensus network, RIATA-HGT retains only HGT events that appear in every set of solutions for every pair of species tree and gene tree. Those events are then added to the species to build the consensus network.

We note here that while offering a simple summary of solutions, this way of computing consensus networks may not be appropriate in general; work is under way to address this issue more properly.

Finally, RIATA-HGT may report '[time violation?]' next to an inferred HGT *X *→ *Y*. If this is the case, this indicates that node *X *lies on the path from *Y *to the root of the species tree. Theoretically, this indicates that two nodes that do not co-exist in time, *X *and *Y *in this case, shared genetic material, and hence the warning of 'time violation.' However, this may be the case simply due to incomplete taxon sampling, as discussed in [19]. Therefore, the warning is issued in this case so as to alert that user that this inferred HGT edge is worth further inspection.

#### Assessing the support of HGT edges

**Figure 8:**
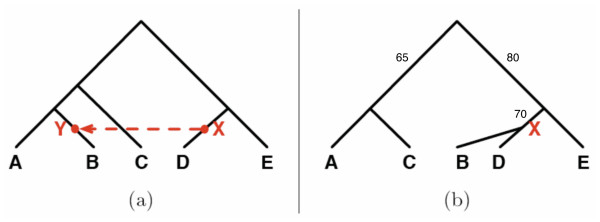
**An illustration of computing the support value of an HGT edge**. An illustration of computing the support value of an HGT edge. In this case, the support of HGT edge *X *→ *Y *added on the species tree (a), is calculated based on the bootstrap of the branches that separate *Y *(or *B*) from *A *in the gene tree (b). The value for the event *X *→ *Y *is 80.

In [37] we proposed a method for assessing the support of HGT edges. Roughly speaking, the support value of HGT edge *X *→ *Y *in the species tree, where *Y*'is the sibling of *Y*, is derived from the bootstrap values of the gene tree branches that separate the clade under *Y *from the clade under *Y*'. The rationale behind the idea is that if *Y*' and *Y *are well separated in the gene trees (i.e., some branches in the path from *Y *to *Y*' have high bootstrap values), HGT is necessary to move *Y *away from *Y*'). For example, the support of HGT edge *X *→ *Y *in Figure 8(a) is calculated based on the bootstrap values of the branches separating *B *from *A *in the gene tree, and it is 80 (which is the maximum bootstrap value of all edges on the path separating *A *and *B *in the gene tree). More technical details can be found in [37].

**Figure 9:**
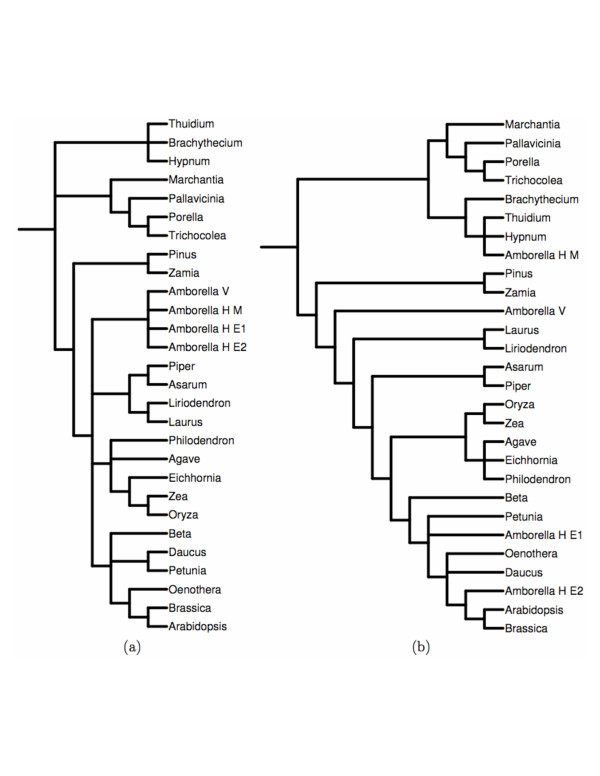
**The cox2 trees**. The species tree (a): (((Pallavicinia, (Porella, Trichocolea)I15)I16, Marchantia)I17, (Thuidium, Brachythecium, Hypnum)I14, (((Amborella_V, Amborella_H_M, Amborella_H_E1, Amborella_H_E2)I9, ((Eichhornia, (Zea, Oryza)I6)I7, Philodendron, Agave)I8, ((Daucus, Petunia)I4, Beta, (Oenothera, (Brassica, Arabidopsis)I2)I3)I5, ((Piper, Asarum)I10, (Liriodendron, Laurus)I11)I1)I12, (Pinus, Zamia)I13)I0)I18; and gene tree (b): ((((((((Petunia, Amborella_H_E1, (((Arabidopsis, Brassica)I2:99.0, Amborella_H_E2), Oenothera, Daucus)), Beta):73.0, ((Agave, Eichhornia, Philodendron), (Oryza, Zea)I6:100.0)), (Asarum, Piper)I10), (Laurus, Liriodendron)I11), Amborella_V), (Pinus, Zamia)I13:72.0), (((Thuidium, Hypnum, Amborella_H_M):91.0, Brachythecium):98.0, (Marchantia, ((Porella, Trichocolea):97.0, Pallavicinia))I17)); for gene *cox2*. Bootstrap values for the branches in the gene tree that are greater than 50.0% are included in the tree Newick representation. The species tree branches do not have bootstrap values.

**Figure 10:**
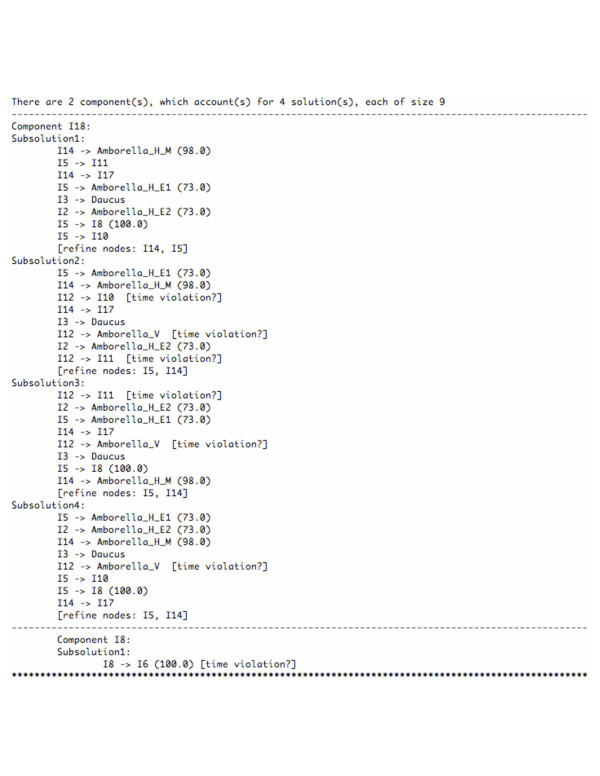
**An example of RIATA-HGT output**. The output of RIATA-HGT on the species tree and *cox2 *gene tree in Figure 9. RIATA-HGT finds 4 solutions, summarized in terms of two components, so that each solution is the union of exactly one subsolution from each component.

Than *et al*. [37] have studied the reliability of this method for assessing the support of HGT edges on various data sets from [38]. In this paper, we illustrate the output of RIATA-HGT on a pair of species/gene trees from [38], as shown in Figure 9. The output of RIATA-HGT on this pair of trees is shown in Figure 10. RIATA-HGT computed four solutions, each of which has nine HGT edges. To allow for a compact representation of the solutions, they are divided into two components (which are computed automatically by RIATA-HGT), and each solution is formed by taking one subsolution from each component. HGT edges for the solutions are divided into two components, which means that a complete solution is formed by taking one solution from each component. Each component is labeled by the name of the internal node that is the root of the clade corresponding to that component. In the case of the solutions presented in Figure 10, each solution contains nine HGT edges, eight of which form a single subsolution in Component I18 and the ninth is the only edge in the only subsolution in Component I8. The value in parentheses next to each HGT edge is its support value computed from the bootstrap values of the gene tree branches (Figure 9(b)). Bergthorsson *et. al*. [38] reported three HGTs involving *Amborella*: one HGT with donor being a species in the *Moss *group (species *Brachythecium*, *Hypnum*, and *Thuidium*, under the internal node I14 in the species tree) and the other two with donors being species in the *Eudicot *group (species *Arabidopsis*, *Beta*, *Brassica*, *Daucus*, *Petunia*, and *Oenothera*, under the internal node I5 in the species tree). The HGT from *Moss *has high SH support value [39]. RIATA-HGT finds this event, I14 → *Amborella_H_M*, with bootstrap value 98.0%. The other two HGT events from *Eudicot *do not have significant SH support values. RIATA-HGT also finds these events, I5 → *Amborella_H_E1 *and I2 → *Amborella_H_E2*. However, their support values are 73.0%, much smaller than that of the event from *Moss*. In addition to these three HGT edges, RIATA-HGT identified six more edges, four out of which had support values smaller than 50.0% (RIATA-HGT does not display support values that are smaller than 50.0%). The HGT edge from Component I8, which is shared among all four solutions, has support value of 100.0%. This edge was not reported in [38]. A similar situation arises with the HGT edge I5 → I8, which is part of the three solutions that contain subsolutions 1, 3, and 4 from Component I18: the HGT edge has support of 100.0%, and was not reported in [38], which may be a reflection that the authors focused only on HGT events involving *Amborella*. The ninth HGT edge in Subsolution2 of Component I18 has support value smaller than 50.0%.

### Other utilities

As evident from the description of the methods above, there are fundamental correlations between phylogenetic trees and networks. Hence, many of the evolutionary network utilities use functionalities from the phylogenetic trees domain, which we have implemented and provide as standalone tools in PhyloNet:

• A tool for computing the maximum agreement subtree (MAST) of a pair of trees using the algorithm of Steel and Warnow [35]. We also extended the algorithm so that it computes *all *MASTs of a pair of tree, and this feature is implemented as well.

• A tool for computing the Robinson-Foulds distance measure between two phylogenetic trees [27].

• A tool for computing the *last common ancestor *(lca) of a set of nodes in a phylogenetic tree.

Additionally, PhyloNet provides an implementation of the parsimony-based method RECOMP of Ruths and Nakhleh [40,41] for detecting interspecific recombination in a sequence alignment.

## Implementation

A major goal for the PhyloNet package was to make its functionality platform-independent and accessible both to end users for data analysis and to researchers designing new computational methods and techniques. In order to encompass as many platforms as possible, PhyloNet was implemented in Java. As a result, any system with the Java 2 Platform (Version 5.0 or higher) installed can run PhyloNet.

PhyloNet can be used in two ways, depending on how the functionality needs to be accessed. A command-line interface exposes all of PhyloNet's tools on a Unix or DOS command-line. Each command accepts input from and writes output to text files. This allows PhyloNet's functionality to be used for manual data analysis or integrated into scripts for performing larger-scale processing. Additionally, a rich and thoroughly documented object model allows the incorporation of any of PhyloNet's functionality into existing Java programs. Also bundled are various programmatic utilities that represent trees, networks, and that read and write these various data structures to and from files.

### The command line interface

PhyloNet has a consistent and easy-to-use command line interface. A detailed discussion of this interface and all available options is available in the documentation that accompanies a download of the tool. Here we provide a brief overview of the design of the command-line tool and the tools that can be accessed. Table 3 lists all the commands that are currently available from the command-line. Each of these commands accepts a set of parameters as command-line arguments. All trees, networks, sequences, and other major data structures are read in either from standard in or from text files. Similarly all results can be written either to standard out or to a desired text file. All trees are read and written in Newick format. Networks are read and written in eNewick format. These design features allow the easy use of PhyloNet for manual data analysis or as a tool used within a larger scripted automated analysis.

**Table 3:** List of tools and their description.

**Tool name**	**Purpose**
charnet	Computing clusters, trees and tripartitions in a network
cmpnets	Computing the distance between two networks
lca	Finding the last common ancestor of a set of nodes
mast	Computing the maximum agreement subtree
netpars	Scoring the parsimony of sequences on a pair of networks
riatahgt	Reconstructing HGT events from a pair of species/gene trees
rf	Computing the Robinson-Foulds tree measure

A table of the tools currently implemented in PhyloNet. With the exception of the three phylogenetic trees tools lca, mast, and rf, all the other tools are for analyzing reticulate evolutionary relationships.

With the exception of the RECOMP tool, all the functionality of PhyloNet is independent of other third party tools. Because RECOMP must compute many trees using Maximum Parsimony trees, this tool requires that PAUP* [42] be installed on the local system. To run a tool in PhyloNet, invoke the executable *.jar *file downloaded from the PhyloNet project homepage:

java -jar phylonet.jar charnet -i net.in -m tree

Here phylonet.jar is the executable jar downloaded from the project homepage (the fle is assumed to be in the current directory where the user invokes this command), charnet is the name of the tool that decomposes the network contained in file net.in into a set of trees. The reference manual included with the executable jar provides very detailed instructions regarding how to run each tool in the PhyloNet package.

### Programmatic interface

Many phylogenetic methods comprise critical, but intermediate, steps in much larger methods. As a result, there is also a need for the functionality in PhyloNet to be available for incorporation into larger programs. As a result, all of PhyloNet's functionality is exposed through an extensive set of Java classes. Each tool is contained within its own Java class and exposes a carefully constructed set of public methods that will be preserved and maintained even as PhyloNet grows. This modular design allows for the easy addition functionality in the future without effecting existing programs that use PhyloNet as a programmatic library. In addition to exposing a consistent API, PhyloNet also provides implementations of the most common phylogenetic data structures: trees and networks. Utility classes are also included that read and write these data structures to and from files. These classes can accelerate not only incorporation of PhyloNet's functionality, but also the development of new phylogenetic functionality within other applications. As PhyloNet grows, programmatic interfaces will be added to provide access to new functionality and tools. Detailed documentation of these libraries is available in JavaDoc form on the PhyloNet website.

## Conclusion

Analyzing and understanding reticulate evolutionary relationships have been hindered by the lack of software tools for conducting these tasks. The proposed software package, PhyloNet, offers an array of utilities to allow for efficient and accurate analysis of such evolutionary relationships. These utilities allow for representing networks in a compact way, characterizing networks in terms of basic building blocks and comparing them based on these characterizations, comparing networks in terms of their fitness to the evolution of a given data set of sequences under the maximum parsimony model, and reconstructing networks from species/gene trees.

The software package will help significantly in analyzing large data sets, as well as in studying the performance of evolutionary network reconstruction methods. Further, the software package offers the novel eNewick format for compact representation of evolutionary networks, a feature that allows for efficient interoperability of evolutionary network software tools.

## Availability and requirements

1. **Project name: **PhyloNet | Phylogenetic Networks Toolkit.

2. **Project home page: **.

3. **Operating system: **Platform independent.

4. **Programming language: **Java.

5. **Other requirements: **Java 1.5, PAUP* (for some applications).

6. **License: **GNU GPL.

7. **Any restrictions to use by non-academics: **The GNU GPL license applies.

## Authors' contributions

All authors contributed equally to the work described in this manuscript.
